# High heterogeneity of malaria transmission and a large sub-patent and diverse reservoir of infection in Wusab As Safil district, Republic of Yemen

**DOI:** 10.1186/s12936-016-1249-y

**Published:** 2016-04-08

**Authors:** Jackie Cook, Lynn Grignard, Samira Al-Eryani, Mustafa Al-Selwei, Abraham Mnzava, Hafed Al-Yarie, Alison Rand, Immo Kleinschmidt, Chris Drakeley

**Affiliations:** MRC Tropical Epidemiology Group, London School of Hygiene and Tropical Medicine, London, UK; Faculty of Infectious and Tropical Diseases, London School of Hygiene and Tropical Medicine, London, UK; Department of Medical Parasitology, Faculty of Medicine and Health Sciences, Sana’a University, Sana’a, Yemen; Malaria Control Programme, Ministry of Public Health and Population, Sana’a, Yemen; Global Malaria Programme, World Health Organization, Geneva, Switzerland

**Keywords:** Malaria transmission, Molecular, Heterogeneity, PCR, Heterozygosity, Multiplicity of infection

## Abstract

**Background:**

Yemen remains the country with the highest malaria transmission within the Arabian Peninsula and a source of imported cases to neighbouring countries.

**Methods:**

This study collected samples from individuals resident in a valley in Western Yemen as a baseline to examine infection prevalence for a future trial. As well as rapid diagnostic test (RDT) and microscopy, a filter paper blood spot was collected for molecular and serological analyses.

**Results:**

Samples were collected from 2261 individuals from 12 clusters across a study area of approximately 100 km^2^. *Plasmodium falciparum* infection prevalence was 12.4, 11.1 and 19.6 % by RDT, microscopy and polymerase chain reaction (PCR), respectively. RDT and microscopy did not detect 45 % of infections present, suggesting many infections were low-density. Infection prevalence and seroprevalence were highly heterogeneous between clusters, with evidence of higher exposure in clusters close to the wadi. The mean multiplicity of infection (MOI) was 2.3 and high heterozygosity and allelic richness were detected.

**Conclusions:**

This highly diverse parasite population suggests a high degree of transmissibility and coupled with the substantial proportion of low-density infections, may pose challenges for malaria control and elimination efforts.

**Electronic supplementary material:**

The online version of this article (doi:10.1186/s12936-016-1249-y) contains supplementary material, which is available to authorized users.

## Background

The Arabian Peninsula, comprising of Bahrain, Kuwait, Oman, Saudi Arabia, Yemen, Qatar and the United Arab Emirates, has made great strides towards malaria elimination over the past 50 years. Of the seven countries in the region, only Saudi Arabia and Yemen still experience ongoing local transmission [1]. Whilst transmission in Saudi Arabia is contained to two south western provinces [2], Yemen struggles with ongoing transmission across the country, with an estimated 25 % of the population at risk of transmission (>1 case per 1000 population) [3].

Yemen is one of the poorest and least developed countries in the Arabian Peninsula and the combination of political unrest and widespread resistance to chloroquine, which was the first-line anti-malarial drug until 2005, resulted in a resurgence of malaria during the 1990s, exacerbated by widespread flooding and unusual rain patterns due to the El Niño climate oscillation [1]. In 2001, a new malaria strategic plan was implemented by the government, which focused on the use of indoor residual spraying (IRS), larviciding and insecticide-treated nets (ITN). In 2009, the first-line drug was switched to artemisinin-based combination therapy (ACT) [3]. Subsequently, malaria incidence appears to have reduced, although data on confirmed cases are sparse [1].

**Fig. 1:**
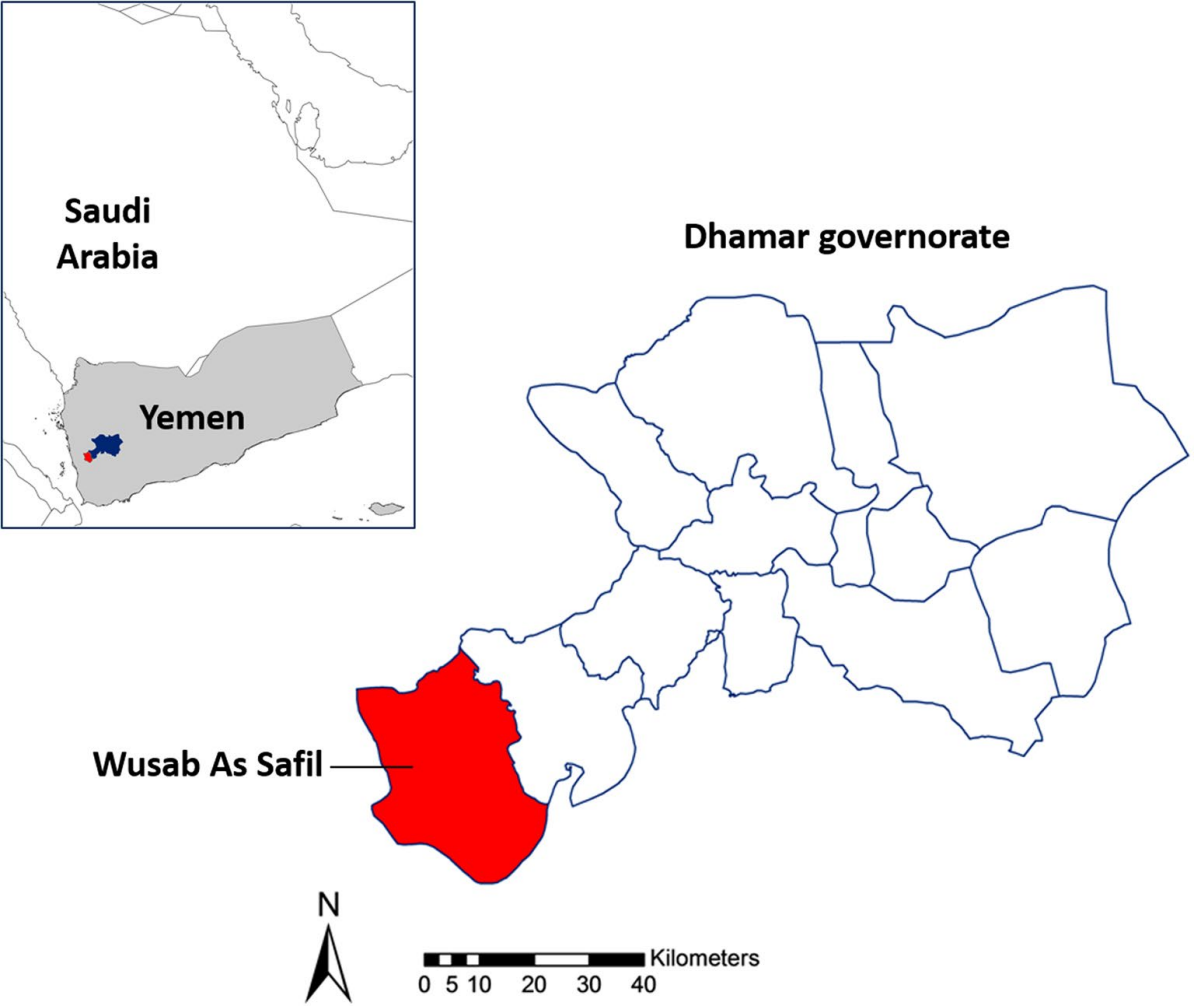
Location of study district, South West Yemen

Wadi Sukhmal is a valley in a mountainous area, located in Wusab As Safil district, approximately 360 km from the capital Sana’a, in the west of the governorate of Dhamar. The district is partially situated in the Tihama Plains which border the Red Sea in Yemen and Saudi Arabia. As an area with ongoing malaria transmission this district was chosen as a site for a wider trial comparing the effects of combining long-lasting insecticide-treated nets (LLIN) and IRS versus LLINs alone. A baseline malaria survey of the population was conducted and filter paper samples collected from residents in order to estimate prevalence and genetic complexity of malaria infections. The genetic complexity and number of clones circulating in the parasite population in an area is often correlated with transmission intensity [4–7]. In addition, filter paper samples were tested for the presence of antibodies to *Plasmodium falciparum* and *Plasmodium vivax* antigens in order to estimate transmission intensity and reconstruct historical transmission patterns in the area. Seroconversion rate and antibody dynamics in a population have been shown to correlate with transmission intensity in a number of settings and can be particularly useful in areas of low transmission [8–10]. All measures were collected as part of both the epidemiological characterization of the study area and as potential endpoints for the intervention trial.

## Methods

### Setting

The study took place in Wusab As Safil district, situated in the Western foothills of Yemen with a population of approximately 150,000 (Fig. 1). Malaria across Yemen is unstable and related to altitude, rainfall and topography. In the study area, where altitude ranges between 601 and 1000 m above sea level, malaria occurs 10 months of the year, although peaks in transmission are seen during and following the rains and is predominantly due to *P. falciparum*. The main vectors are *Anopheles arabiensis* and *Anopheles sergenti* which are thought to breed in the fresh-water wadi (riverbed/stream) in the valley. The majority of the district is agricultural and is made up of mountains and valleys. The size of the study area was approximately 100 km^2^.

### Survey and sample collection

Samples were collected as part of a baseline survey for a larger trial to assess the effects of combining LLIN and IRS compared with LLIN alone. Clusters were selected from villages near Wadi Sukhmal, which was chosen due to high malaria incidence recorded by local health facilities. The survey took place in 12 clusters in the wadi that were randomized after stratification by distance to the wadi and baseline prevalence of infection, which was measured by rapid diagnostic test (RDT) and microscopy in October 2011. The average population in the study clusters was 800 (range: 337–1677). Clusters were separated by at least 500 m. Filter paper samples were collected during a second baseline survey which took place in March 2012, prior to the trial intervention. Households were randomly sampled from within each cluster. Every child aged 15 and under who was present at the time of visit had a finger prick blood sample taken for subsequent microscopy, as well as for an RDT (Carestart HRP-2, a *P. falciparum* specific test), and a filter paper for molecular and serological analyses.

### Laboratory methods

#### DNA extraction and PCR amplification

DNA was extracted from three punches of 3 mm diameter from each filter paper sample (corresponding to approximately 10 µl of blood) using the Chelex method, as documented previously [8]. The extracted DNA (5 µl) was amplified by 18S ribosomal DNA (rDNA) nested PCR [9] using primers listed in Table 1. Subsequently species specific (*P. falciparum, Plasmodium malariae, P. vivax*) nested PCR was performed (Table 1). *P. malariae* and *P. vivax* PCR were only run on samples from three selected clusters due to suspected low prevalence of these species. Nested PCR products were analysed on 1.5 % agarose gels.

**Table 1:** Primers and sequences used for nested PCR

Snounou PCR		
Primary	rPLU6	5′TTAAAATTGTTGCAGTTAAAACG3′
	rPLU5new	5′CYTGTTGTTGCCTTAAACTTC3′
*P. falciparum*	rFAL1	5′TTAAACTGGTTTGGGAAAACCAAATATATT3′
	rFAL2	5′ACACAATAGACTCAATCATGACTACCCGTC3′
*P. malariae*	rMAL1	5′ATAACATAGTTGTACGTTAAGAATAACCGC3′
	rMAL2	5′AAAATTCCCATGCATAAAAAATTATACAAA3′
*P. vivax*	rVIV1	5′CGCTTCTAGCTTAATCCACATAA3′
	rVIV2	5′ACTTCCAAGCCGAAGCAAAGAAA 3′
MSP2 PCR		
Primary	S2-Fw	5′GAA GGT AAT TAA AAC ATT GTC3′
	S3-REv	5′GAG GGA TGT TGC TGC TCC ACA G3′
Nested	S1_Tail_	5′GCT TAT AAT ATG AGT ATA AGG AGA A3′
	M5	5′-6FAM-GCA TTG CCA GAA CTT GAA3′
	N5	5′-VIC-CTG AAG AGG TAC TGG TAG A3′

#### MSP-2 genotyping

The *msp2* genotyping was adapted from a published protocol [10]. Briefly, 5 µl of Chelex-extracted DNA was added to the primary PCR containing 500 nM forward and reverse primers (Table 1). The nested PCR contained 300 nM forward primer S1_Tail_ and 300 nM fluorescently labelled reverse primers M5 (Fc27 family specific) and N5 (3D7 allelic family specific).

Samples were analysed together with 500-ROX size standard (Applied Biosystems) at the MRC Genomics Core Facility (Imperial College, Hammersmith Campus). The data was analysed using the GeneMapper Software (Applied Biosystems). All detected peaks were sorted by size and divided into three base pair bins to call the alleles.

#### Enzyme-linked immunosorbent asssay (ELISA)

Filter paper samples were stored at 4 °C with desiccant until processed, as previously described [11]. Samples were tested at a final serum dilution equivalent of 1:1000 for human immunoglobulin G antibodies against *P*. *falciparum* and *P. vivax* merozoite surface protein 1_19_ (MSP-1_19_) and *P. vivax* apical membrane antigen-1 (AMA-1) and 1:2000 for antibodies against *P. falciparum* AMA-1 using ELISA methods described in detail previously [11]. Optical Densities (OD) were normalized against the standard curve on each plate to account for differences between ELISA batches. ELISA results for each antigen were classified as positive and negative using a mixture model, as previously described [11]. Individuals were classed as positive if they had antibodies to either antigen.

#### Data analyses

Statistical analysis was performed using STATA 13 (StataCorp, Texas, US). RDT and microscopy results were compared using Cohen’s kappa. Sensitivity and specificity of RDT and microscopy results were calculated using *P. falciparum* PCR as the gold standard. Standard errors of all prevalence estimates were adjusted using robust variance estimates [12] as implemented in the *svy* commands in Stata [13] to account for between cluster variation of responses, using household or cluster as primary sampling unit when presenting cluster-level or overall prevalence, respectively. To examine the effect of age on MOI and prevalence, age data was split into 0–1 year olds, 1–5 year olds and 5–15 year olds. The non-parametric Spearman’s Rho test was used to measure associations at an individual and cluster level.

#### Genetic diversity and population structure

The multiplicity of infection was defined as the number of different *msp*-*2* alleles detected in one sample. The expected heterozygosity index (He), which measures the diversity at a locus, was calculated using the Excel Microsatellite Toolkit software [14]. Heterozygosity can range from 0 to 1, where low heterozygosity indicates inbreeding, or a small population diversity, whilst a high heterozygosity indicates the presence of genetic variability. Allelic richness, which is the mean number of alleles per locus normalized to allow for sample size among populations, was calculated using FSTAT software. In addition, the fixation index (Wright’s F statistic), a measure of population variance, was calculated using FSTAT allowing 55,000 permutations.

#### Serological analyses

Seroprevalence to *P. falciparum* and *P. vivax* antigens was modelled against age using a simple reversible catalytic model as previously described [15]. In order to examine whether a change in transmission intensity had occurred, profile likelihood plots were generated to see whether fitting a model with two seroconversion rates may be more appropriate than a model with a single seroconversion rate [16]. A likelihood ratio test was used to determine which model fitted the data best.

## Ethics approval

Ethics approval was obtained from the National Committee for Health Research, Ministry of Health, Yemen. Informed consent was obtained from all participants or their guardians, prior to the collection of samples.

## Results

### Study population

A total of 2261 filter paper samples were collected, with an average of 185 children per cluster (range 163–216) aged <16 years. The median age was 6 years (25–75 range: 3–9) and 53 % were female. 1341 (60 % of study population) individuals were classified as living within 500 m of the wadi.

### *Plasmodium* infections detected by RDT and microscopy

*Plasmodium falciparum* prevalence was 12.4 % (10.3–14.6) (n = 281) and 11.1 % (9.2–13.1) (n = 237) by RDT and microscopy, respectively, with a Cohen’s kappa of 0.83. Of the 281 RDT positive individuals, 62 were not positive by microscopy, whilst of 237 microscopy *P. falciparum* positive individuals, 16 were not positive by RDT. In addition, microscopy identified 19 *P. malariae* infections (overall prevalence of 0.8 %).

### *Plasmodium* infections detected by PCR

PCR results were successfully obtained for 2181 samples. Of these, 19.6 % (95 % CI 17.3–21.9) (n = 430) were positive for *P. falciparum*. Further analysis for malaria species was performed in three clusters (clusters 5, 10 and 12) in which four *P. vivax* infections were found [all in cluster 12, (2.2 %)] and 17 *P. malariae* infections were found [in clusters 5 (2 %), 10 (6 %) and 12 (0.6 %)].

### Prevalence of low-density malaria infections and spatial heterogeneity

**Fig. 2:**
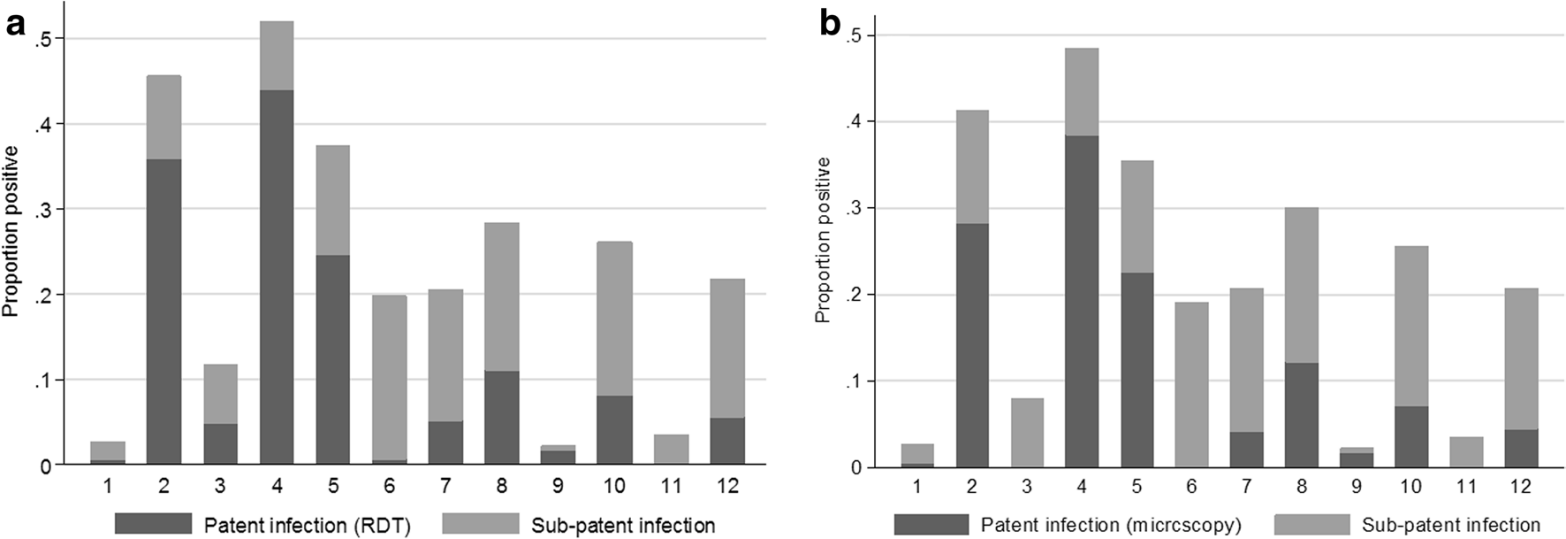
Proportion of patent (detectable by **a** RDT and **b** microscopy) and sub-patent (detected by PCR) *P. falciparum* infections by cluster

Using *P. falciparum* PCR as the gold standard, RDT had a sensitivity of 45.8 % (41.0–50.6) and a specificity of 95.4 % (94.3–96.3) and *P. falciparum* microscopy had a sensitivity of 41.6 % (36.9–46.4) and a specificity of 97.1 % (96.3–97.9), although sensitivity for both diagnostic methods varied substantially by cluster, ranging from 0 % in cluster 1 to 78.3 % in cluster 4. In general, sensitivity was higher in clusters of higher prevalence, suggesting more of the infections were high density in these areas (Fig. 2). Approximately 45 % of *P. falciparum* infections present were not detected by RDT and microscopy. This did not vary by age group. Only 5 of 11 microscopy positive *P. malariae* infections that were tested with PCR were confirmed as positive.

**Fig. 3:**
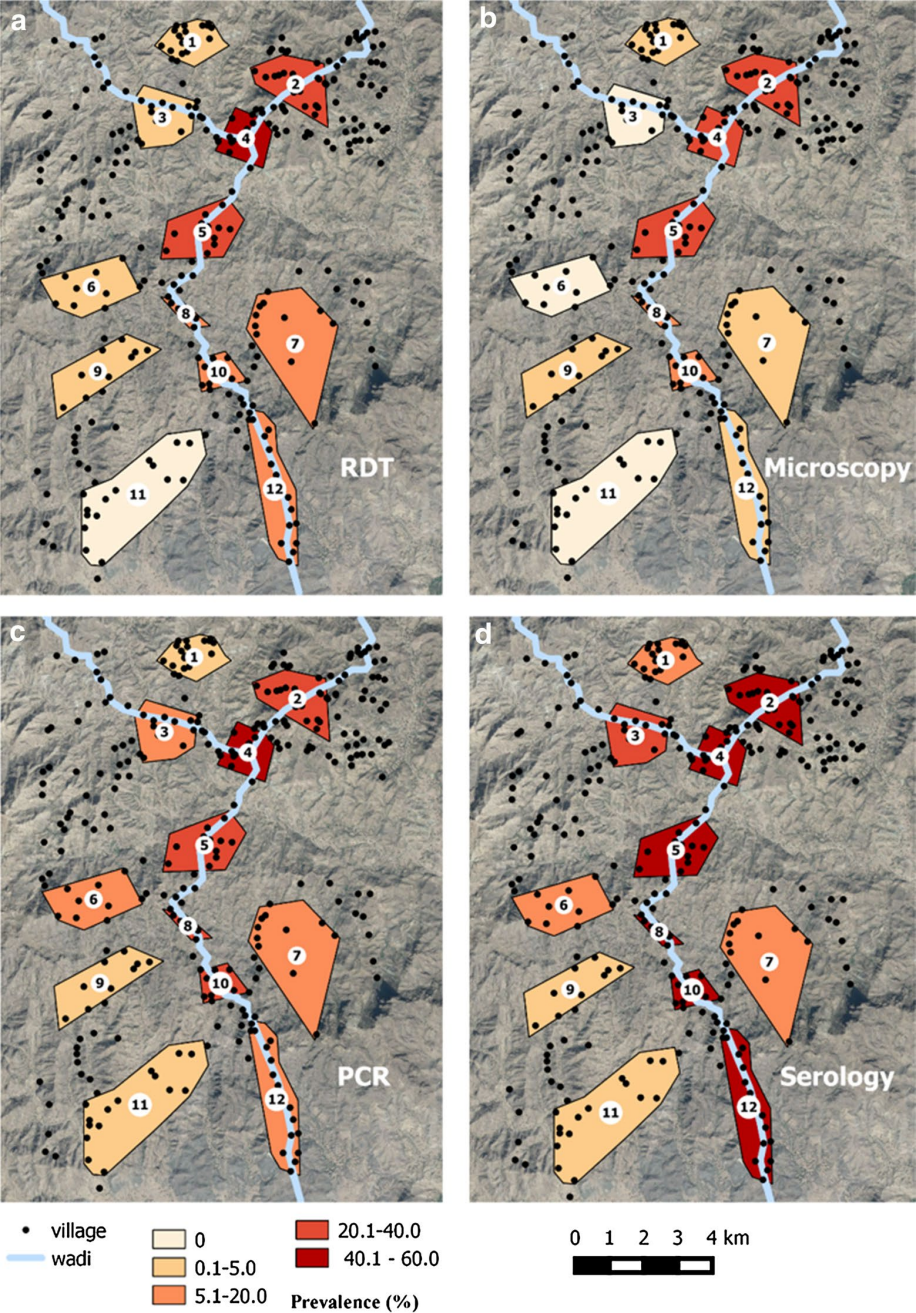
Prevalence for **a** RDT **b** Microscopy **c** PCR and **d** seroprevalence by cluster

**Fig. 4:**
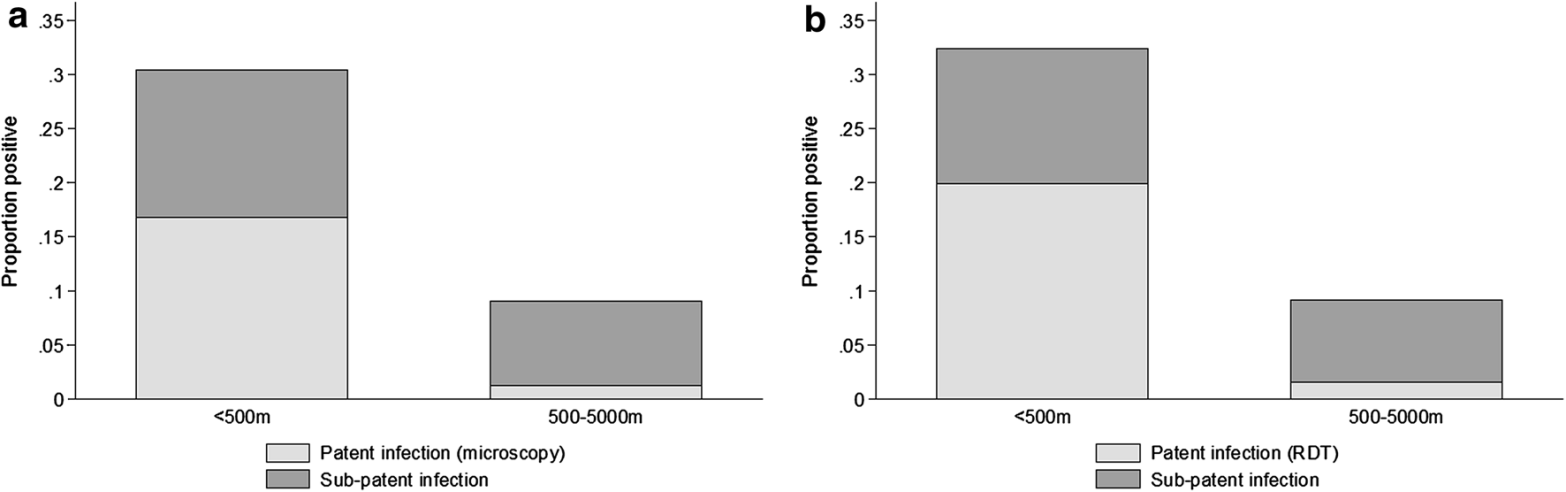
Proportion of patent (detectable by **a** RDT and **b** microscopy) and sub-patent (detected by PCR) *P. falciparum* infections by distance to the wadi

As with the other parasite prevalence measures, parasite prevalence by PCR ranged substantially by cluster (Figs. 2, 3). The highest prevalence was detected in Cluster 4 (46.0 %, 92/200) with cluster 2 also being high (37.6 %, 78/206). The lowest prevalence was in clusters 1 and 9, where prevalence was 2.2 % (4/185 and 4/182 respectively) (Table 2). Parasite prevalence by PCR was highest in clusters that were located on the wadi (26.8 %, 355/1325), compared with clusters located >500 m from the wadi (8.5 %, 72/850 p < 0.001). This difference was even more pronounced with RDT or microscopy parasite measures (19.9 and 1.6 % for RDT and 16.8 and 1.3 % for microscopy) (Fig. 4).

**Table 2:** *P. falciparum* prevalence as measured by RDT, microscopy, PCR and antibodies to either *P. falciparum* MSP-1 or AMA-1, by cluster

Cluster	Tested	RDT			Pf microscopy			Pf PCR			Pf serology		
	N	+ve	%	95 % CI	+ve	%	95 % CI	+ve	%	95 % CI	+ve	%	95 % CI
1	185	1	0.5	−0.5, 1.6	1	0.5	−0.5, 1.6	4	2.2	−1.5, 5.8	17	9.2	3.2, 15.2
2	216	77	35.6	24.7, 46.6	61	27.8	18.6, 37	78	37.6	27.4, 47.7	121	58.5	49.6, 67.5
3	187	9	4.8	1.6, 8	0	0	0, 0	15	8	3, 13	60	32.1	24.7, 39.5
4	200	88	44	37.1, 50.9	77	38.5	31.8, 45.2	92	46	38.8, 53.2	118	59	51.8, 66.2
5	195	48	24.6	15.6, 33.6	44	22.6	15.1, 30.1	53	27.2	20.4, 34	110	56.4	47.1, 65.8
6	163	1	0.6	−0.6, 1.8	0	0	0, 0	31	19.1	11.9, 26.3	22	13.6	7.2, 20
7	173	9	6	1.5, 10.5	7	4.7	1.1, 8.3	27	18	11, 25	32	17.3	6.4, 28.2
8	180	20	10.9	3.7, 18.1	22	10.3	3.7, 17	40	23	16.2, 29.7	74	42.5	35.1, 50
9	182	3	1.6	−0.1, 3.4	3	1.6	−0.1, 3.4	4	2.2	0.3, 4.1	9	4.9	1.4, 8.5
10	184	15	8.2	3.2, 13.1	13	7.1	3.1, 11	44	23.9	17.5, 30.3	80	43.5	34.5, 52.5
11	178	0	0	0, 0	0	0	0, 0	6	3.5	1.2, 5.8	6	3.5	0.4, 6.7
12	179	10	5.6	1.7, 9.4	8	4.5	0.6, 8.3	33	18.4	11.9, 25	76	42.5	32.9, 52
Total	2220	281	12.7	10.5, 14.8	236	10.5	8.7, 12.3	427	19.6	17.3, 21.9	725	33.0	30.0, 36.0

### Seroprevalence to *P. falciparum* antigens

Seroprevalence for *P. falciparum* antigens was 23.1 % (n = 453), 21.2 % (n = 471) and 31.8 % (n = 729) for MSP-1_19_, AMA-1, or either antigen, respectively. As with the parasitological results, seroprevalence ranged substantially between clusters, with clusters 2 and 5 having the highest prevalence (58.5 %, 121/216 and 56.4 %, 110/195 for either antigen, respectively) whilst clusters 1 and 11 were low (9.2 %, 17/185 and 3.5 %, 6/178 for either antigen, respectively) (Table 2).

### Seroprevalence to *P.vivax* antigens

Whilst *P. vivax* specific PCR was only performed on samples from three clusters, all samples were tested on *P. vivax* MSP-1_19_ and AMA-1 antigens to look for *P. vivax* specific antibodies. Seroprevalence was 1.4 % (n = 30), 2.2 % (n = 47) and 3.2 % (n = 75) for MSP-1_19_, AMA-1, or either antigen respectively. None of the four *P. vivax* infections detected by PCR were antibody positive to either antigen. No children under 1 year of age were seropositive for any *P. vivax* antigens, with highest seroprevalence detected in 12–15 year olds (5.7 %). As with the other prevalence measures, seroprevalence to *P. vivax* antigens was highest in the clusters located closest to the wadi (4.5 %, 60/1341), compared to those located further away (1.7 %, 15/880). Sixty-five percent (n = 49) of *P. vivax* serological positive samples were also positive for *P. falciparum* antibodies.

### Historical patterns of transmission patterns elucidated from serological data

**Fig. 5:**
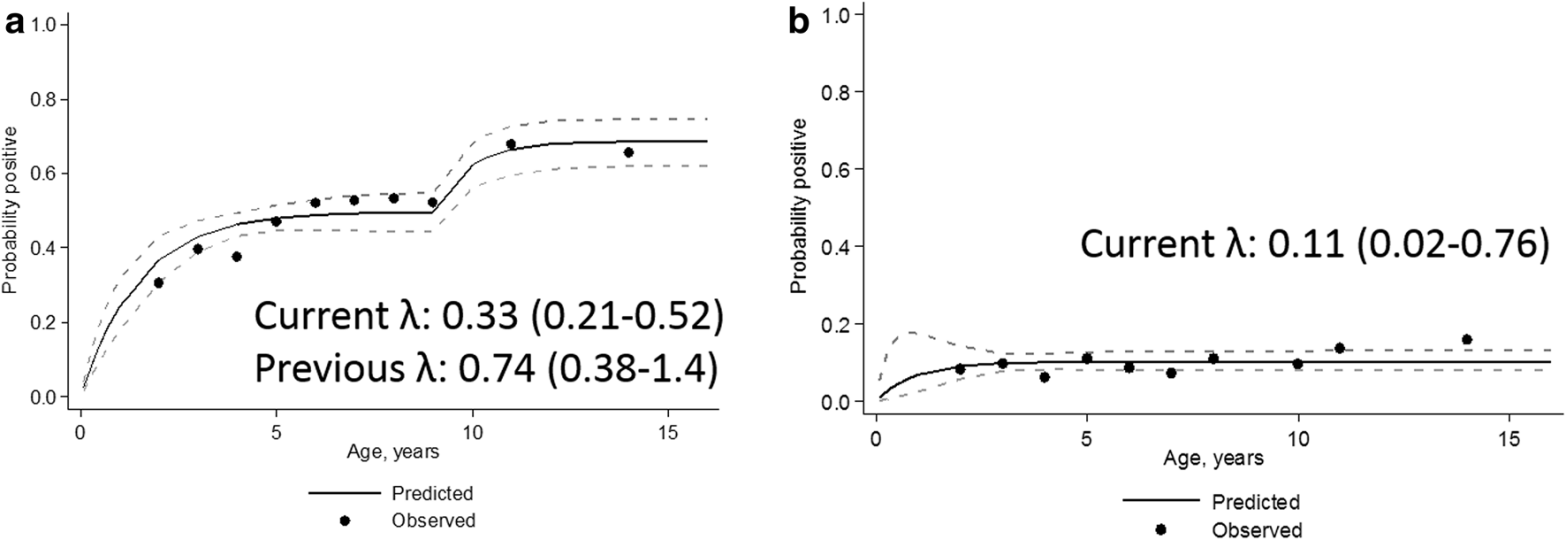
Seroprevalence curves to either *P. falciparum* antigen for **a** people living within 500 m of the wadi and **b** further than 500 m from the wadi. *Black circles* represent actual data, plotted at median percentiles for age and *black line* represents the results from a reversible catalytic model. In the clusters close to the wadi, there was evidence for a change in transmission 9 years prior to the survey—this dataset has been fitted allowing for two lambdas

Age seroprevalence curves for responses to either *P. falciparum* antigen were modelled using a simple reversible catalytic model for clusters within 500 m of the wadi and for those further away. The seroconversion rate in the clusters on the wadi was double that of the ones further away (λ 0.22 and λ 0.11 respectively, equivalent to EIRs of approximately 15 and 3 [15]). Profile likelihood analysis showed no evidence for a recent change in transmission in the low transmission clusters. However, in clusters close to the wadi, a model with a change in force of infection 9 years previously fit the data best (p < 0.001) (Fig. 5). In these clusters, seroconversion rates for children under the age of nine were half that of those over the age of nine (λ 0.33 and λ 0.74 in children under and over nine, respectively).

### Genetic diversity of *Plasmodium falciparum* infections

**Fig. 6:**
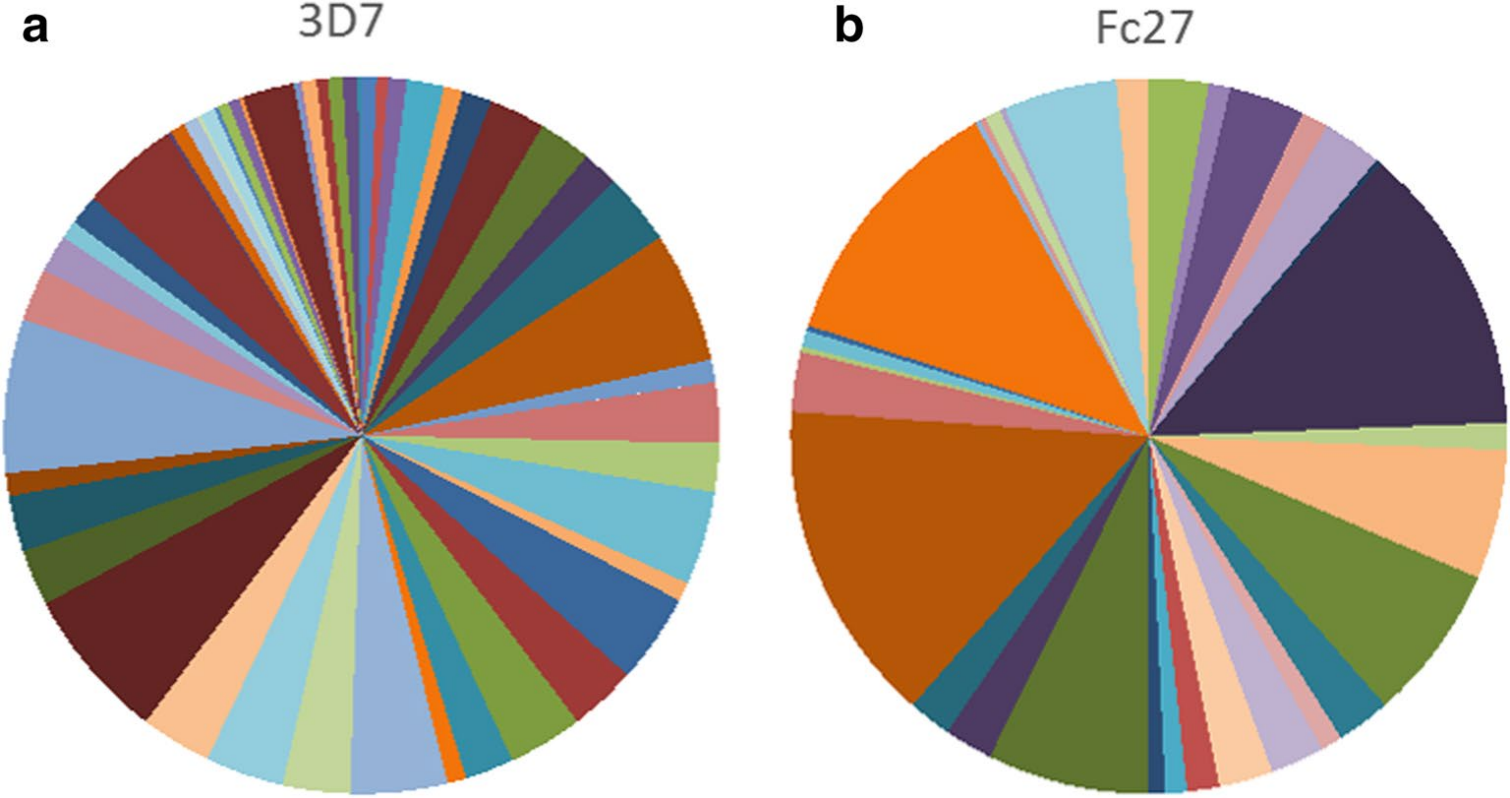
Frequency of **a** 3D7 and **b** Fc27 allelles in *P. falciparum* samples, Yemen

*msp2* markers were amplified from 339 (79 %) of the *P. falciparum* positive samples (N = 430). A total of 84 different *msp2* alleles were successfully genotyped, ranging in size from 205 to 609 base-pairs. Of these, 62 % (n = 52) and 38 % (n = 32) belonged to the 3D7 and Fc27 families respectively (Fig. 6). The frequency of individual 3D7 alleles was low: all had a frequency below 5 %. 63 % (n = 211) of infections were made up of 2 or more alleles (range 1–10). The mean MOI was 2.3, with the highest MOI detected in cluster 5 (3.1) (Table 3). The MOI decreased slightly with age group, although this was not significant. Nor was there a significant relationship between clusters close or far from the wadi or between clusters themselves; all clusters had a number of infections consisting of more than one allele. The estimated heterozygosity was above 0.9 in all clusters apart from cluster 11 (0.75) (Table 3). Allelic richness was 11.7 and ranged from 3.9 to 11.6 by cluster. The overall fixation index for the 12 clusters was 0.029, which is suggestive of little genetic differentiation and frequent gene flow between all clusters.

**Table 3:** Multiplicity of infection (MOI), allelic richness and heterozygosity at the msp2 alleles in *P. falciparum* infections in Yemen

Cluster	No. of PCR positives	MOI (range)	Allelic richness	Heterozygosity (He)	Number of alleles sampled
1	4	1.75 (1–3)	5	0.905	5
2	57	1.67 (1–6)	10	0.94	34
3	9	2.22 (1–4)	8.5	0.937	12
4	76	2.43 (1–7)	11.7	0.973	51
5	50	3.06 (1–8)	11.3	0.969	41
6	17	2.71 (1–7)	7.8	0.878	17
7	14	1.64 (1–5)	9.1	0.94	15
8	34	2.65 (1–10)	9.9	0.941	34
9	1	2.00 (2)			
10	40	1.88 (1–4)	11	0.967	35
11	5	1.40 (1–2)	3.9	0.75	4
12	29	2.28 (1–8)	10.5	0.954	28

### Correlations between metrics of transmission

At a cluster level, the parasite prevalence measures were strongly associated with *P. falciparum* seroprevalence (ρ 0.94 p < 0.001, ρ 0.86 p < 0.001 and ρ 0.90 p < 0.001 for RDT, microscopy and PCR, respectively). All *P. falciparum* prevalence and seroprevalence measures were strongly correlated with allelic richness (ρ 0.90 p < 0.001, ρ 0.85 p = 0.001, ρ 0.85 p = 0.001, ρ 0.96 p < 0.001 for RDT, microscopy, PCR and seroprevalence respectively) and expected heterozygosity (ρ 0.85, p < 0.001, ρ 0.83 p = 0.002, ρ 0.75 p = 0.008, ρ 0.91 p < 0.001). As previously described, expected heterozygosity was strongly correlated with allelic richness (ρ 0.97 p < 0.001). Interestingly, cluster MOI was not significantly associated with any other measures. Correlations are shown in Additional file 1.

## Discussion

Malaria remains a significant public health problem in Yemen. This study demonstrates the wide range of malaria infection prevalence and exposure in communities separated by short distances in a single valley in Western Yemen using a variety of metrics. Parasite prevalence was notably high in some clusters (up to 46 %), although heterogeneity was marked. A large proportion of sub-patent infections were detected, indicating that diagnosis using RDTs or microscopy would leave a significant number of infections untreated. Moreover, the highly diverse nature of the parasite population in this study area is suggestive of a high degree of transmissibility and may pose a barrier for malaria control efforts in the region.

The marked heterogeneity of infection prevalence in this setting is surprising given the short distance between the clusters sampled i.e. prevalence between cluster 1 (2 %) and 4 (46 %) despite only being 2 km apart (Fig. 3). Heterogeneity in infection has been reported in many settings [17, 18] although rarely to the same extreme as seen in this setting over such short distances [19, 20]. Variation in infection prevalence can be due to variability in risk factors such as use of interventions, poor quality housing or location relative to mosquito breeding sites. A clear risk factor in this setting was the wadi, with clusters located within 500 m of the wadi demonstrating consistently higher prevalence. The riverine environment is likely to be a breeding site for the primary malaria vector in the area, *An. arabiensis* [21] and one explanation for the high hetereogenity is that the range of the mosquitoes is relatively restricted and that there is limited mosquito movement between clusters. A recent study conducted in different districts in Yemen also found a range of parasite prevalences, although over a wider distance than is reported here [22]. In that study, house structure and occupation were most likely to influence prevalence. This information was not collected in the current study and is a limitation in maximizing the findings from the data collected.

The high proportion of sub-patent infections poses challenges for malaria control and elimination efforts. This study observed discordance between microscopy (which is the main diagnostic in Yemen) and RDT, as well as approximately 45 % of infections being missed by these methods. Whilst low parasite density infections are likely to be less infectious to mosquitoes [23], they still represent a risk for onward transmission and such a large reservoir is likely to play a role in transmission [24]. The proportion of sub-patent infections was higher further from the wadi, in the lower transmission clusters; 82 % of infections were sub-patent in the clusters further from the wadi, compared to 38 % in those close to the wadi. This is consistent with observations from large-scale comparison of PCR and RDT/microscopy which found the highest levels of sub-patent infections in low transmission settings [24]. As molecular diagnostics have become more sensitive, it has become clear that low-density infections are often present in high numbers, even in areas of relatively low transmission intensity [23, 25, 26]. The duration of these low-density infections, which are often asymptomatic, has been shown to be much longer than thought [27], although data is relatively sparse. Chronic, low-density infections are a barrier for malaria elimination in areas where *Anopheles* mosquitoes are still present.

The high diversity of the parasite population in this region was surprising although is consistent with other studies from Yemen [28, 29]. Previous molecular studies have noted that a highly diverse parasite population tends to be associated with higher malaria transmission [30]. In this study area, where there is a range of transmission intensity, the genetic diversity was high, even in lower transmission clusters. This apparent discordance may occur in areas where transmission has only recently reduced, such that there is only a subtle change in diversity [31] or when a change has not yet become detectable [32, 33]. However, the serological results suggest that transmission has been consistently low in the clusters further from the wadi, with no evidence for a recent change in transmission. High heterozygosity was also detected in nearby areas in a previous study [29] and is suggestive of a large parasite population circulating in Yemen. This could be influenced by population movement in the region, with the high level of parasite diversity in this study being similar to that seen in highly endemic African populations [10, 34].

In this study, there was no association found between MOI and transmission intensity. Whilst many studies have shown that MOI is higher in areas of greater transmission intensity [10, 20], there have been other studies that demonstrate no correlation [35]. It is possible that the timing of the survey may have influenced this with a study in Senegal showing that MOI increased during the transmission season [36].

Serological data indicates a significant difference in exposure to infection between children born 9 years previously compared with those born more recently, with older children experiencing approximately double the transmission rate. This effect is most apparent in the clusters located within 500 m of the wadi. This difference may be due to an age related behavioural pattern, for example if children over ten spend more time outdoors and/or in high-risk areas, as has been seen observed in South East Asia [37, 38] and South America [39, 40].

Alternatively, this difference could be indicative of a fall in transmission 9 years previously and in this case enhanced efforts of the malaria control programme, initiated in 2002. However, the fact that samples were only collected from those <16 years means it is difficult to fully reconstruct historical transmission patterns using serological data and it is possible a different pattern would have been apparent if samples from all ages had been examined.

Despite fewer samples being tested for *P. vivax* and *P. malariae* parasites using PCR, data suggest that *P. vivax* prevalence is extremely low with no infections detected by microscopy, in line with the national control programme estimates [3]. Seroprevalence to *P. vivax* antigens was below 10 % in all clusters, with only a small number of children with high antibody responses to either *P. vivax* antigen. Several *P. malariae* infections were identified in the clusters that were tested. This had also been detected in previous molecular studies in Yemen [41]. The presence of multiple *Plasmodium* species has implications for diagnosis, with RDT and microscopy lacking sensitivity for species other than *P. falciparum*.

As well as being informative for transmission intensity levels, understanding the genetic diversity of parasites in Yemen may help elimination programmes in the region to accurately classify a case as imported or locally acquired. Such a diverse parasite population may pose challenges for the effectiveness of malaria vaccines, which need to target the most common allelic families present in a region [42]. As has been seen in other settings [20, 28], 3D7 alleles were more prevalent in this setting than the Fc27 family.

## Conclusions

This study used a variety of metrics to identify extreme and relatively focal heterogeneity of malaria infection prevalence over small distances in the foothills of Yemen, including a high proportion of sub-patent infections. The parasite population was highly diverse, even in clusters of low prevalence, with transmission focused close to the wadi. Identifying risk factors for high prevalence, such as proximity to mosquito breeding sites or high proportions of the population travelling to high transmission areas, will enable intervention campaigns to target villages appropriately and may result in a more cost-effective approach to reduce transmission in Yemen.

## Additional file

10.1186/s12936-016-1249-y A) Prevalence of *P. falciparum* PCR and RDT, microscopy and seroprevalence data by cluster B) Prevalence of *P. falciparum* PCR and multiplicity of infection, allelic richness and heterozygosity. Quadratic fits are also shown on the graphs.

## References

[1] Snow RW, Amratia P, Zamani G, Mundia CW, Noor AM, Memish ZA (2013). The malaria transition on the Arabian Peninsula: progress toward a malaria-free region between 1960–2010. Adv Parasitol.

[2] Coleman M, Al-Zahrani MH, Coleman M, Hemingway J, Omar A, Stanton MC (2014). A country on the verge of malaria elimination–the Kingdom of Saudi Arabia. PLoS One.

[3] WHO (2013). World Malaria Report: Yemen country profile.

[4] Arnot DE (2002). The influence of the genetic complexity of *Plasmodium falciparum* infections on the epidemiology of malaria. Trans R Soc Trop Med Hyg.

[5] Anderson TJ, Haubold B, Williams JT, Estrada-Franco JG, Richardson L, Mollinedo R (2000). Microsatellite markers reveal a spectrum of population structures in the malaria parasite *Plasmodium falciparum*. Mol Biol Evol.

[6] Hoffmann EH, da Silveira LA, Tonhosolo R, Pereira FJ, Ribeiro WL, Tonon AP (2001). Geographical patterns of allelic diversity in the *Plasmodium falciparum* malaria-vaccine candidate, merozoite surface protein-2. Ann Trop Med Parasitol.

[7] Babiker HA, Lines J, Hill WG, Walliker D (1997). Population structure of *Plasmodium falciparum* in villages with different malaria endemicity in east Africa. Am J Trop Med Hyg.

[8] Plowe CV, Djimde A, Wellems TE, Diop S, Kouriba B, Doumbo OK (1996). Community pyrimethamine-sulfadoxine use and prevalence of resistant *Plasmodium falciparum* genotypes in Mali: a model for deterring resistance. Am J Trop Med Hyg.

[9] Snounou G, Viriyakosol S, Zhu XP, Jarra W, Pinheiro L, do Rosario VE (1993). High sensitivity of detection of human malaria parasites by the use of nested polymerase chain reaction. Mol Biochem Parasitol.

[10] Schoepflin S, Valsangiacomo F, Lin E, Kiniboro B, Mueller I, Felger I (2009). Comparison of *Plasmodium falciparum* allelic frequency distribution in different endemic settings by high-resolution genotyping. Malar J..

[11] Corran PH, Cook J, Lynch C, Leendertse H, Manjurano A, Griffin J (2008). Dried blood spots as a source of anti-malarial antibodies for epidemiological studies. Malar J..

[12] Rao JNKS, Scott AJ (1981). The analysis of categorical data from complex sample surveys: Chi squared tests for goodness-of-fit and independence in two-way tables. J Am Statist Assoc..

[13] Statacorp: Stata: Release 13. Statistical software College Station, TX 2013.

[14] http://www.animalgenomics.ucd.ie/sdepark/ms-toolkit/.

[15] Drakeley CJ, Corran PH, Coleman PG, Tongren JE, McDonald SL, Carneiro I (2005). Estimating medium- and long-term trends in malaria transmission by using serological markers of malaria exposure. Proc Natl Acad Sci.

[16] Cook J, Kleinschmidt I, Schwabe C, Nseng G, Bousema T, Corran PH (2011). Serological markers suggest heterogeneity of effectiveness of malaria control interventions on Bioko Island, equatorial Guinea. PLoS One.

[17] Alemu K, Worku A, Berhane Y (2013). Malaria infection has spatial, temporal, and spatiotemporal heterogeneity in unstable malaria transmission areas in northwest Ethiopia. PLoS One.

[18] Jorgensen P, Nambanya S, Gopinath D, Hongvanthong B, Luangphengsouk K, Bell D (2010). High heterogeneity in *Plasmodium falciparum* risk illustrates the need for detailed mapping to guide resource allocation: a new malaria risk map of the Lao People’s Democratic Republic. Malar J..

[19] Machault V, Gadiaga L, Vignolles C, Jarjaval F, Bouzid S, Sokhna C (2009). Highly focused anopheline breeding sites and malaria transmission in Dakar. Malar J..

[20] Barry AE, Schultz L, Senn N, Nale J, Kiniboro B, Siba PM (2013). High levels of genetic diversity of *Plasmodium falciparum* populations in Papua New Guinea despite variable infection prevalence. Am J Trop Med Hyg.

[21] al-Maktari MT, Bassiouny HK (1999). Bionomics of anopheline vectors in Zabid District, Al-Hodeidah Governorate, Republic of Yemen. East Mediterr Health J..

[22] Bamaga OA, Mahdy MA, Mahmud R, Lim YA (2014). Malaria in Hadhramout, a southeast province of Yemen: prevalence, risk factors, knowledge, attitude and practices (KAPs). Parasit Vectors..

[23] Bousema T, Okell L, Felger I, Drakeley C (2014). Asymptomatic malaria infections: detectability, transmissibility and public health relevance. Nat Rev Microbiol.

[24] Okell LC, Bousema T, Griffin JT, Ouedraogo AL, Ghani AC, Drakeley CJ (2012). Factors determining the occurrence of submicroscopic malaria infections and their relevance for control. Nat Commun..

[25] Golassa L, Enweji N, Erko B, Aseffa A, Swedberg G (2013). Detection of a substantial number of sub-microscopic *Plasmodium falciparum* infections by polymerase chain reaction: a potential threat to malaria control and diagnosis in Ethiopia. Malar J..

[26] Harris I, Sharrock WW, Bain LM, Gray KA, Bobogare A, Boaz L (2010). A large proportion of asymptomatic Plasmodium infections with low and sub-microscopic parasite densities in the low transmission setting of Temotu Province, Solomon Islands: challenges for malaria diagnostics in an elimination setting. Malar J..

[27] Ashley EA, White NJ (2014). The duration of *Plasmodium falciparum* infections. Malar J..

[28] Al-abd NM, Mahdy MA, Al-Mekhlafi AM, Snounou G, Abdul-Majid NB, Al-Mekhlafi HM (2013). The suitability of *P. falciparum* merozoite surface proteins 1 and 2 as genetic markers for in vivo drug trials in Yemen. PLoS One.

[29] Al-Hamidhi S, Mahdy MA, Al-Hashami Z, Al-Farsi H, Al-mekhlafi AM, Idris MA (2013). Genetic diversity of *Plasmodium falciparum* and distribution of drug resistance haplotypes in Yemen. Malar J..

[30] Arnot D (1998). Unstable malaria in Sudan: the influence of the dry season. Clone multiplicity of *Plasmodium falciparum* infections in individuals exposed to variable levels of disease transmission. Trans R Soc Trop Med Hyg.

[31] Sisya TJ, Kamn’gona RM, Vareta JA, Fulakeza JM, Mukaka MF, Seydel KB (2015). Subtle changes in *Plasmodium falciparum* infection complexity following enhanced intervention in Malawi. Acta Trop.

[32] Gunawardena S, Ferreira MU, Kapilananda GM, Wirth DF, Karunaweera ND (2014). The Sri Lankan paradox: high genetic diversity in *Plasmodium vivax* populations despite decreasing levels of malaria transmission. Parasitology.

[33] Yuan L, Zhao H, Wu L, Li X, Parker D, Xu S (2013). *Plasmodium falciparum* populations from northeastern Myanmar display high levels of genetic diversity at multiple antigenic loci. Acta Trop.

[34] Mwingira F, Nkwengulila G, Schoepflin S, Sumari D, Beck HP, Snounou G (2011). *Plasmodium falciparum**msp1*, *msp2* and *glurp* allele frequency and diversity in sub-Saharan Africa. Malar J..

[35] Mara SE, Silue KD, Raso G, N’Guetta SP, N’Goran EK, Tanner M (2013). Genetic diversity of *Plasmodium falciparum* among school-aged children from the Man region, western Cote d’Ivoire. Malar J..

[36] Vafa M, Troye-Blomberg M, Anchang J, Garcia A, Migot-Nabias F (2008). Multiplicity of *Plasmodium falciparum* infection in asymptomatic children in Senegal: relation to transmission, age and erythrocyte variants. Malar J..

[37] Cook J, Speybroeck N, Sochanta T, Somony H, Sokny M, Claes F (2012). Sero-epidemiological evaluation of changes in *Plasmodium falciparum* and *Plasmodium vivax* transmission patterns over the rainy season in Cambodia. Malar J..

[38] Erhart A, Ngo DT, Phan VK, Ta TT, Van Overmeir C, Speybroeck N (2005). Epidemiology of forest malaria in central Vietnam: a large scale cross-sectional survey. Malar J..

[39] Ferreira IM, Yokoo EM, Souza-Santos R, Galvao ND, Atanaka-Santos M (2012). Factors associated with the incidence of malaria in settlement areas in the district of Juruena, Mato Grosso state Brazil. Cien Saude Colet..

[40] Parker BS, Paredes Olortegui M, Penataro Yori P, Escobedo K, Florin D, Rengifo Pinedo S (2013). Hyperendemic malaria transmission in areas of occupation-related travel in the Peruvian Amazon. Malar J..

[41] Al-Mekhlafi AM, Mahdy MA (2010). A AA, Fong MY. Molecular epidemiology of Plasmodium species prevalent in Yemen based on 18s rRNA. Parasit Vectors..

[42] Takala SL, Plowe CV (2009). Genetic diversity and malaria vaccine design, testing and efficacy: preventing and overcoming ‘vaccine resistant malaria’. Parasite Immunol.

