# Supplementary figures and images for: High heterogeneity of malaria transmission and a large sub-patent and diverse reservoir of infection in Wusab As Safil district, Republic of Yemen
Source: Malar J. 2016 Apr 8;15:193. doi: 10.1186/s12936-016-1249-y (PMC4826523; doi:10.1186/s12936-016-1249-y)

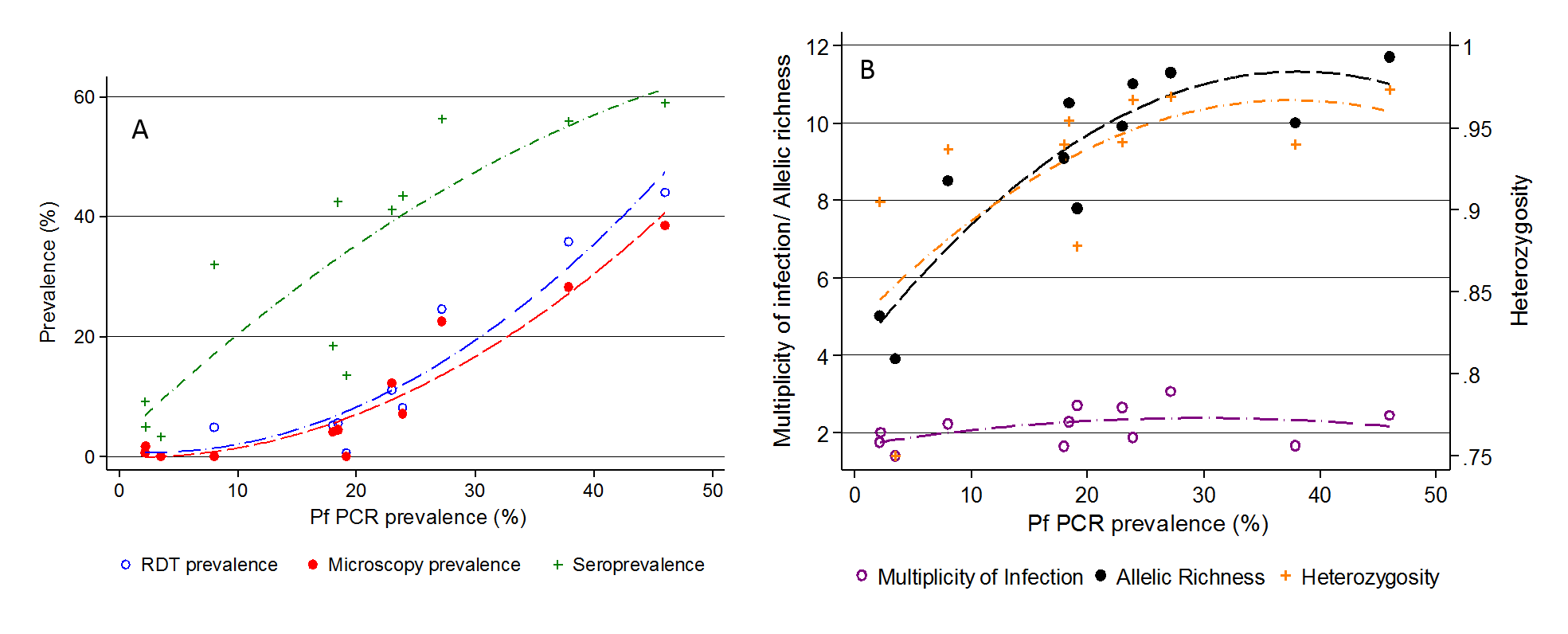

Supplement: Supplementary file 1 — 10.1186/s12936-016-1249-y A) Prevalence of P. falciparum PCR and RDT, microscopy and seroprevalence data by cluster B) Prevalence of P. falciparum PCR and multiplicity of infection, allelic richness and heterozygosity. Quadratic fits are also shown on the graphs. [file 12936_2016_1249_MOESM1_ESM.png]
